# Reconstitution of membrane contact by unilamellar vesicles

**DOI:** 10.52601/bpr.2023.230011

**Published:** 2023-08-31

**Authors:** Shulin Li, Min Zhang, Liang Ge

**Affiliations:** 1 State Key Laboratory of Membrane Biology, Beijing 100101, China; 2 Tsinghua-Peking Center for Life Sciences, Beijing 100084, China; 3 School of Life Sciences, Tsinghua University, Beijing 100084, China; 4 School of Pharmaceutical Sciences, Tsinghua University, Beijing 100084, China

**Keywords:** Membrane contacts, Organelle interaction network, Giant unilamellar vesicle (GUV), Liposome, ER-Golgi intermediate compartment (ERGIC), ER-exit sites (ERES), COPII, TMED9, SEC12, Autophagy, Autophagosome

## Abstract

Eukaryotic cells compartmentalize diverse biochemical functions within organelles defined by intracellular membranes. Recent focus has intensified on studying the interactions among organelles and the role of membrane contacts in maintaining cellular balance. While analyzing these contacts mainly involves fluorescence and electron microscopy, as well as biochemical cell fractionation, understanding their mechanisms and responses to genetic and environmental changes remains challenging. Here we describe an approach employing *in vitro* reconstitution of membrane contacts using unilamellar vesicles. This technique offers insights into contact mechanisms when combined with established methods like fluorescence imaging and mass spectrometry, potentially deepening our understanding of membrane contacts and organelle networks.

## INTRODUCTION

Eukaryotic cells harbor a complex endomembrane system, historically studied to distinguish distinctive traits of individual compartments. As comprehension of organelle networks deepens and imaging techniques advance, the significance of organelle interactions has gained prominence (Giordano *et al.* 2013; Hamasaki *et al.* 2013; Nascimbeni *et al.* 2017; Scorrano *et al.* 2019; Zhao *et al.* 2018). Hence, dissecting the molecular mechanisms of membrane contacts is pivotal for comprehending organelle interactions and the broader endomembrane system. The *in vitro* reconstitution utilizing unilamellar vesicles emerges as a potent tool for investigating these contacts. This article delineates an *in vitro* giant unilamellar vesicle (GUV) tether assay, *in vitro* SAR1 transactivation, and membrane interaction assays to dissect contact mechanisms, exemplified by the ER-Golgi intermediate compartment (ERGIC)–ER-exit sites (ERES) contact facilitated by TMED9 and SEC12 for autophagosome biogenesis (Lamb *et al.* 2013; Li *et al.* 2021, 2022; Mizushima *et al.* 2008; Puri and Rubinsztein 2021).

The GUV tether assay employs fluorescently labeled tether proteins, enabling the precise visualization of membrane contacts. Through density gradient centrifugation, non-GUV-conjugated proteins are removed. Co-incubated GUVs, examined via fluorescence microscopy, reveal contacts, aiding the analysis of their dynamics and biophysical traits. The introduction of active components like cytosol or cell-fraction, followed by mass spectrometry, identifies essential factors for contact formation. *In vitro* reconstitution directly elucidates a factor's role, complementing *in vivo* approaches such as the *in vitro* GUV tether assay combined with genetic screening, for comprehensive contact mechanism dissection.

Procedures for membrane interaction assays and subsequent SAR1 transactivation are also outlined. These assays encompass SAR1 purification, small unilamellar vesicle (SUV) preparation, and investigations into membrane interactions and SAR1 transactivations. Such methods illuminate cellular membrane dynamics and membrane contacts, contributing to deeper insights in the field.

## MATERIALS

### Biological materials

• *E. coli* BL21 strain

### Reagents

• 1-palmitoyl-2-oleoyl-glycero-3-phosphocholine (POPC; Avanti Polar Lipids)

• 1,2-dioleoyl-sn-glycero-3-phosphoethanolamine(DOPE; Avanti Polar Lipids)

• 1-palmitoyl-2-oleoyl-sn-glycero-3-phospho-L-serine (POPS; Avanti Polar Lipids)

• DOPE-rhodamine (Avanti Polar Lipids)

• PE-biotin (Avanti Polar Lipids)

• Cholesterol (Avanti Polar Lipid)

• 2% 1,2-dioleoyl-sn-glycero-3-phosphoethanolamine-N-[4-(p-maleimidophenyl) butyramide] (PE-MPB; Avanti Polar Lipids)

• 2% 1,2-dioleoyl-sn-glycero-3-[(N-(5-amino-1-carboxypentyl) iminodiacetic acid) succinyl] (DGS-NTA; Avanti Polar Lipids)

• FITC-TM9CT peptide and FITC-TM9CT (m4) peptide (Scilight Biotechnology LLC)

• OptiPrep

• pGEX4T1 vector

• Lysozyme (Sigma)

• Dithiothreitol (DTT; Genview)

• Protease inhibitors (Roche)

• GTP Guanosine triphosphate (GTP; Solarbio)

• Glutathione agarose beads (GE)

• AminoLink-beads (Sigma)

• Streptavidin agarose beads (GE)

### Equipment and software

• Glass slides

• Glass syringe (HEMILTON)

• 100 nm pore size polycarbonate film

• Nitrogen gas

• Liquid nitrogen

• Confocal dish

• Centrifuge (Beckman Optima MAX-XP)

• Laser scanning confocal microscope (Olympus FV3000)

• ImageJ software

## PROCEDURE

### Step 1: *In vitro* GUV tether assay

#### 1.1 Lipid mixture preparation

(A) Weigh the appropriate amounts of POPC, DOPE, POPS, and cholesterol as per the molar ratio (4∶2.5∶2.5∶1) specified in the protocol.

i. For the ERGIC-GUVs, add 2% PE-MPB to the lipid mixture and mix thoroughly.

ii. For the ERES-GUVs, add 2% DGS-NTA, 0.1% DOPE-rhodamine, and 1% PE-biotin to the lipid mixture and mix thoroughly.

**[Tips]** The composition of lipids here can be modified according to the membrane components under study.

(B) Dissolve the lipids in an appropriate solvent ( *e*. *g*., chloroform) to create a lipid solution.

(C) Mix the lipids thoroughly using gentle agitation or vertexing to ensure a homogeneous mixture.

#### 1.2 GUV preparation

(A) Using 200 μL of 1% agarose to coat one clean glass slide overnight, meanwhile, the other glass slide was coated by using 7 μg of streptavidin (dissolved in 200 μL ddH _2_O).

(B) Pipette the lipid solution onto the center of the slide.

(C) Using a glass syringe ( *e*. *g*., HEMILTON), carefully spread the lipid solution across the glass slide to form a thin lipid film.

(D) Allow the sample to dry with a gentle stream of nitrogen gas for approximately 3 h. The nitrogen gas helps to evaporate the solvent and form a lipid film.

#### 1.3 GUV suspension

(A) Gently add 200 μL of phosphate-buffered saline (PBS) to the glass slide containing the dried lipid film.

(B) Incubate the slide with PBS for 10 min to rehydrate the lipids and form GUVs.

**[Tips]** (1) GUVs are prepared using the hydration method here, and other methods such as electroformation can also be employed for their preparation. (2) After the specific GUVs are prepared, they can be observed using a microscope to assess their size and yield, ensuring they meet the requirements for subsequent experiments.

#### 1.4 Peptides or protein crosslinking and attachment ( *Fig. 1*)

**Figure 1:**
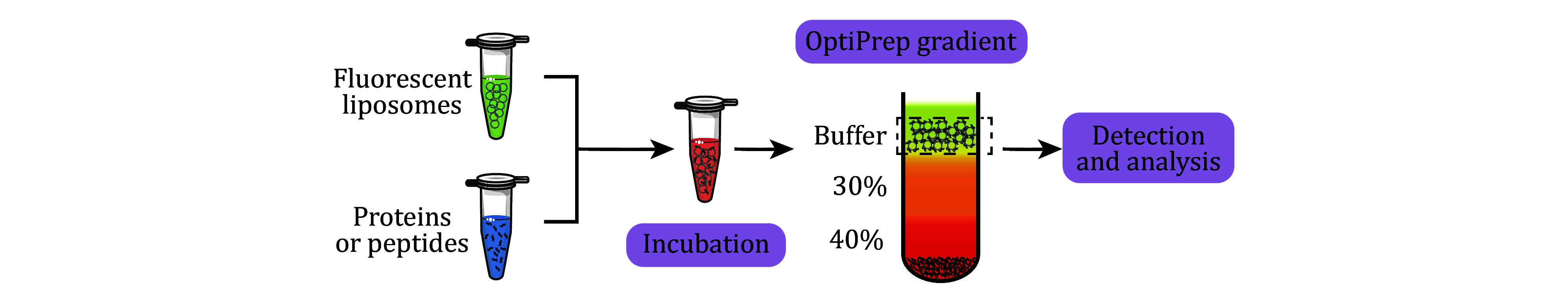
Peptide or protein crosslinking and removal of free peptides or proteins

(A) If using FITC-TM9CT or FITC-TM9CT (m4) peptides, or His-SEC12 protein, add them to the respective lipid mixtures (300 μL solution containing 3 μg of peptides or proteins).

(B) Perform the crosslinking or attachment reaction for 30 min with gentle rotation at room temperature. The rotation ensures even distribution and interaction of peptides/proteins with GUVs.

**[Tips]** After the preparation of GUVs, all subsequent steps should be carried out gently and with great care to minimize the risk of damaging the GUVs.

#### 1.5 Removal of free peptides or proteins ( *Fig. 1*)

(A) To remove free peptides or proteins, perform a membrane flotation procedure as follows:

i. For each 300 μL solution, add 300 μL of 50% OptiPrep (diluted in PBS).

ii. Carefully overlay the mixture with 480 μL of 20% OptiPrep and 90 μL of PBS.

iii. Centrifuge the mixture at 100,000 *g* for 2 h to separate proteoliposomes from free peptides or proteins.

iv. Collect the 150 μL top fraction, which contains the proteoliposomes.

#### 1.6 ERGIC-GUVs and ERES-GUVs mixing

(A) Mix the "ERES-GUVs" and "ERGIC-GUVs" at a 1∶1 volume ratio to combine the modified vesicles.

**[Tips]** The specific ratio of GUVs at this stage needs to be adjusted based on the yield of GUVs and experimental requirements. The final ratio of GUVs should mimic, to the greatest extent possible, the proportion of two types of biological membranes under physiological conditions.

(B) Incubate the mixed vesicles for an additional 30 min at room temperature.

#### 1.7 Streptavidin-coated glass slides incubation

(A) Add the mixed GUVs to a streptavidin-coated glass slide.

(B) Incubate the dish for 20 min to allow binding of the biotinylated GUVs to the streptavidin-coated surface.

#### 1.8 Imaging and quantification ( *Fig. 2*)

**Figure 2:**
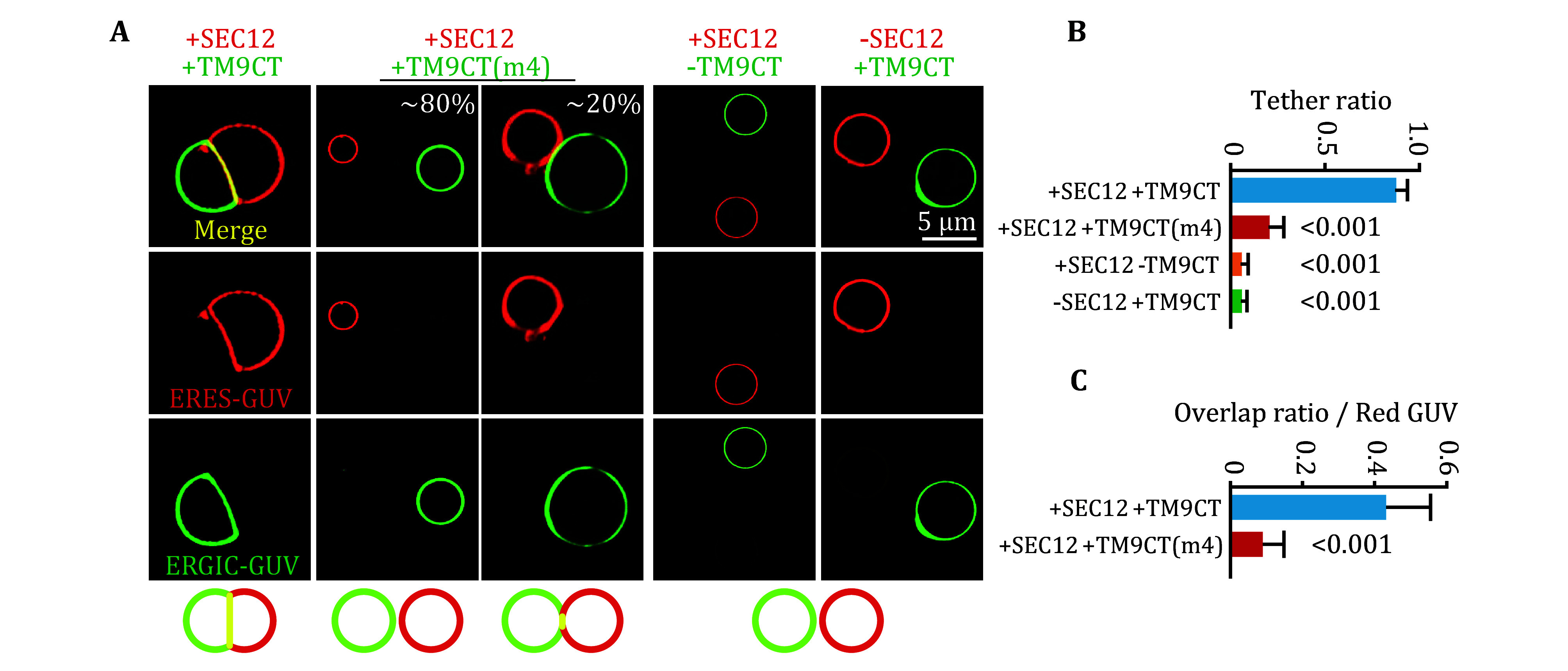
*In vitro* GUV tether assay. **A** Fluorescence images of contact formation between “ERES-GUVs” and “ERGIC-GUVs” with indicated proteins or peptides. **B**, **C** Quantification of tether/contact ratio (ratio of ERES-GUV in contact with ERGIC-GUV, **B**) and overlap ratio (overlap area to ERES-GUV area, **C**), as shown in Panel A. Error bars represent standard deviations of >150 GUVs from three independent experiments (>50 GUVs per experiment). *P* value was obtained from two-tailed *t*-test ( Li *et al.* 2022)

(A) Capture images of the GUVs using a laser scanning confocal microscope ( *e*. *g*., Olympus FV3000).

(B) Use ImageJ to quantitatively analyze the results.

**[Tips]** This may involve measuring vesicle sizes, fluorescence intensities, or other relevant parameters.

### Step 2: *In vitro* membrane interaction assay and *in vitro* transactivation assay of SAR1

#### 2.1 Protein purification

(A) Clone SAR1-BFP, SAR1-T39N-BFP, and BFP genes into the pGEX4T1 vector.

(B) Express proteins in *E. coli* BL21 at 22 °C for 5 h.

(C) Lyse bacteria with 0.5 mg/mL lysozyme and lysis buffer on ice for 0.5 h.

**[Recipe]** Lysis buffer: 50 mmol/L Tris/HCl, pH 8.0, 5 mmol/L EDTA, 150 mmol/L NaCl, 10% glycerol, plus 0.3 mmol/L DTT and protease inhibitors.

(D) Sonicate, centrifuge at 20,000 *g* 4 °C for 1 h, incubate with glutathione agarose beads at 4 °C for 2 h, and wash beads with wash buffer.

**[Recipe]** Wash buffer: PBS with 0.1% Tween 20.

(E) Elute proteins with elution buffer.

**[Recipe]** Elution buffer: 50 mmol/L Tris, pH 8.0, 250 mmol/L KCl, 25 mmol/L glutathione.

(F) Freeze and store purified proteins at –80 °C.

**[Tips]** The protein can be replaced with the desired protein based on experimental requirements.

#### 2.2 Preparation of SUVs

(A) Prepare the lipid mixture as described in the protocol.

(B) Spread the lipid mixture in a thin film on a clean glass slide; dry the lipid mixture with the nitrogen stream and further dry at 37 °C for 1 h.

**[Tips]** Ensure complete evaporation of the solvent.

(C) Suspend lipid film in PBS, then freeze-thaw ten times alternately between liquid nitrogen and a 42 °C water bath.

(D) Extrude lipid film twenty times through a 100-nm pore-size polycarbonate film to produce SUVs.

(E) Incubate FITC-TM9CT peptides with SUVs (300 μL solution contains 3 μg peptides), crosslink for 30 min with rotation at room temperature, and perform membrane flotation as described in Step 1.5 to produce ERGIC-SUVs.

#### 2.3 In vitro membrane interaction assay ( *Fig. 3*)

**Figure 3:**
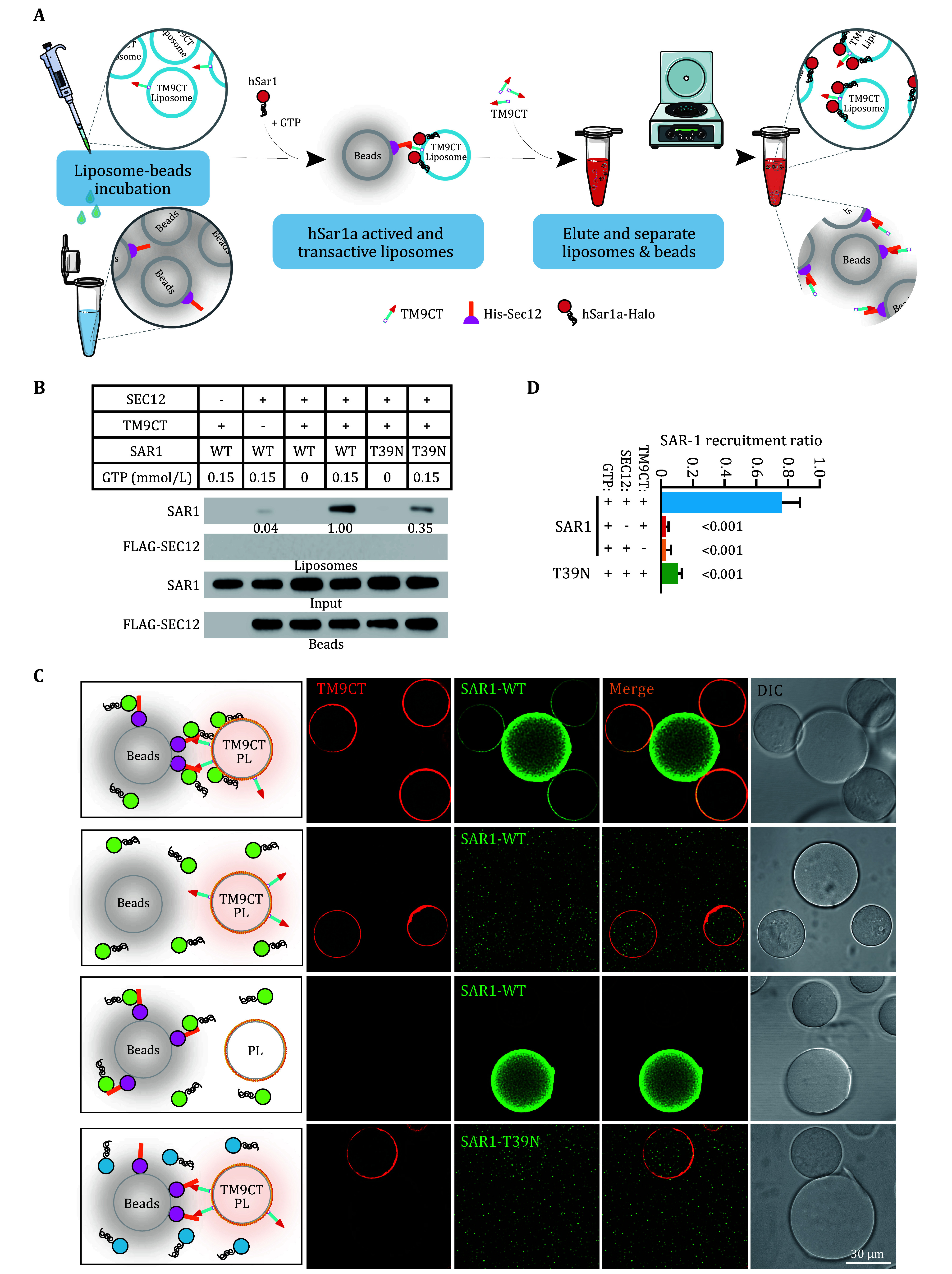


**Figure 3:** *In vitro* membrane interaction assay and *in vitro* transactivation assay of SAR1. **A** The schematic diagram of the *in vitro* membrane interaction assay. **B** Immunoblot showing the transactivation of SAR1 on the liposome via SEC12-bound beads. Beads with or without SEC12 were incubated with liposomes with or without TMED9-CT, and indicated variants of SAR1 in the presence or absence of GTP. After the reaction, the liposomes were isolated followed by immunoblot. Quantification was based on the ratio of SAR1 with the group of highest transactivation (with SEC12, TM9CT, SAR1, and 0.15 nmol/L GTP set as 1.00). The blots are representative of at least three independent experiments. **C** Fluorescence imaging showing the recruitment of SAR1-BFP to the TMED9-CT-labeled liposomes attached to streptavidin agarose beads. Beads with or without SEC12 were incubated with beads coated with control liposomes or TMED9-CT. Indicated SAR1-BFP variants with GTP were incubated with the indicated combination of beads. Confocal imaging was performed to analyze the recruitment of SAR1-BFP to the liposomes on the beads that were in contact with beads containing SEC12. **D** Quantification of SAR1 recruitment ratio as shown in Panel C. Error bars represent standard deviations of >150 beads with liposomes from three independent experiments (>50 beads with liposomes per experiment). *P* value obtained from two-tailed *t*-test (Li *et al.* 2022)

(A) Incubate ERGIC-SUVs with SEC12-crosslinked AminoLink-beads (Covalently crosslink the protein onto the beads according to the instructions provided) overnight at 4 °C.

(B) Wash SEC12 beads three times with PBS, add 50 μg/mL SAR1-BFP or SAR1-T39N-BFP and GTP, and incubate at room temperature for 30 min.

(C) Wash beads-SUVs three times with PBS.

(D) Elute by incubating with 0.5 mg/mL of the TMED9-CT peptides for 30 min at room temperature followed by 1500 *g* centrifugation to separate ERGIC-SUVs and SEC12 beads, and perform immunoblot.

#### 2.4 *In vitro* *transactivation assay of SAR1* ( *Fig. 3*)

(A) Incubate ERGIC-SUVs with Streptavidin agarose beads overnight at 4 °C with slow rotation.

(B) Wash beads three times with PBS.

(C) Prepare reaction mixes with 0.15 mmol/L GTP, SEC12 beads (described above), and respective proteins (according to the experimental setup) at a final concentration of 50 μg/mL in a final volume of 200 µL.

(D) Transfer the reaction mixture to a confocal dish and incubate for 1 h at 30 °C in the dark.

(E) Acquire confocal images with 60× objective, and process using ImageJ.

## PERSPECTIVES

Membrane contacts denote the close juxtaposition of two organelle membranes, emerging as pivotal elements for cellular harmony (Eisenberg-Bord *et al.* 2016; Gatta and Levine 2017; Perez-Sancho *et al.* 2016; Simmen and Tagaya 2017; Wu *et al.* 2018). Although initially glimpsed through electron microscopy in the 1950s (Bernhard and Rouiller 1956; Copeland and Dalton 1959), these sites were overshadowed due to their ephemeral nature and heterogeneous presence among cell types. Presently, the intricate task of visualizing and isolating these contacts to decipher their functions and responses to genetic and environmental shifts persists. Characterizing their constituents and roles faces complexities in monitoring their fluid dynamics. Our study simplifies the intricacies of membrane contacts and introduces tools for their scrutiny. In tandem with proteomic and lipidomic techniques, our approach holds promise in uncovering supplementary protein or lipid factors that regulate the established connectors between these membranes. This offers insights into the assembly of protein complexes and the pathways governing these regulatory processes. Incorporating optical tweezers and advanced imaging refines our grasp of the underlying biophysical mechanisms. Amid the evolving landscapes of biochemistry and cell biology, these methodologies exhibit the potential to untangle the core principles of membrane contacts. Nonetheless, our method primarily centers on predetermined contacts, with limited utility for unknown instances, necessitating validation under physiological conditions. Transmembrane proteins can help establish contacts between membranes. However, the integration of integral membrane proteins onto GUVs has proven challenging. By further refining the system to enable the incorporation of transmembrane protein functionality, we can expand its utility for studying membrane contacts.

## Conflict of interest

Shulin Li, Min Zhang and Liang Ge declare that they have no conflict of interest.
